# Research on region selection super resolution restoration algorithm based on infrared micro-scanning optical imaging model

**DOI:** 10.1038/s41598-021-82119-1

**Published:** 2021-02-02

**Authors:** Jian Chen, Yan Li, LiHua Cao

**Affiliations:** Changchun Institute of Optics, Fine Mechanics and Physics, Chinese Academy of Sciences, Changchun, 130033 Jilin China

**Keywords:** Imaging and sensing, Electrical and electronic engineering

## Abstract

With spring up of infrared imaging related industry, infrared imaging technology has become mainstream development direction of intelligent photoelectrical detection due to its good concealment, wide detection range, high positioning accuracy, long distant penetration, light weight, little volume, low power dissipation and high solidity. However, the features of infrared dim-small target image such as less details and low SNR become bottleneck of infrared image application. How to enhance imaging effect of infrared dim-small target becomes research hotspot. Starting from the point of ‘restoration as foundation’, the theory and technology of infrared dim-small target super-resolution restoration by utilizing the theory and technology of super-resolution restoration are explored in this paper. This paper mainly focuses on the research of super-resolution restoration algorithm of infrared dim-small target based on infrared micro-scanning optical model. Aiming at solving super-resolution restoration problem of infrared dim-small target, the traditional super-resolution restoration algorithm is optimized and the improved algorithm is proposed. Meanwhile, infrared micro-scanning optical model is introduced to break theoretical limit of simple image processing algorithm. And the performance of infrared image super-resolution restoration is improved.

## Introduction

Projection Onto Convex Sets (POCS) is intuitive and simple coded. It uses prior knowledge set intersection to search solution space. It has inherent advantages to lead into prior knowledge. Meanwhile the complex image degradation model can be built. So it becomes one of most popular spatial super resolution restoration methods^1^. However, POCS has inherent shortcomings such as extremely sensitive to noise. The restoration quality of POCS will seriously decline by large noise. In application the low resolution images usually contain various noise of transmission system and coherent noise. So POCS cannot directly apply to super resolution restoration of viewing images. Considering the condition of noise interference, a POCS super resolution restoration algorithm based on BM3D^2^ was proposed. This arithmetic can effectively depress noise. The low resolution image of low SNR (Signal to Noise Ratio) can still get better restoration effect.

The traditional super resolution restoration algorithm generates one high resolution image by using multi frame low resolution image sequences in time^3^. There are two problems by using this method: one is the time lag of multi frame low resolution image sequences in time, the other is spatial displacement of multi frame low resolution image sequences in time. There exists error in the input source of super resolution restoration. The research of past super resolution restoration has entered bottleneck, and the restoration performance is difficult to further improve. In this paper, the reflective infrared micro-scanning optical model is proposed, and the multi frame low resolution image sequences are generated by using this model. There are no time lap and spatial displacement of the multi frame low resolution image sequences. So the error in the input source of super resolution restoration is eliminated. Furthermore, the combination of infrared micro-scanning optical model and super resolution restoration breaks through the theoretical limit comparing with single imaging algorithm and the performance of super resolution restoration is greatly improved. Considering the case of real-time processing, this paper proposes a POCS super resolution restoration algorithm based on region selection, which can distinguish target region and background and greatly reduces computation time.

## Reflective infrared micro-scanning optical model

**Figure 1:**
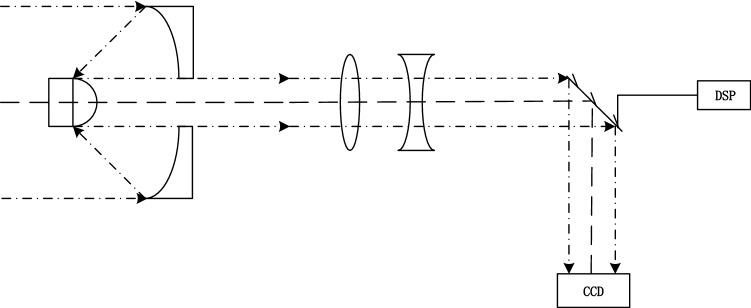
Reflective infrared micro-scanning optical model.

The reflective infrared micro-scanning optical model designed in this paper can achieve controllable micro-scanning at any step length, thus providing low resolution images with corresponding frames for various super magnification imaging. The real time infrared images are obtained through the high speed vibration of high speed mirror. This model can obtain high speed and real time multi frame sub-pixel displacement images. The reflective infrared micro-scanning optical model is shown in Fig. 1.

**Figure 2:**
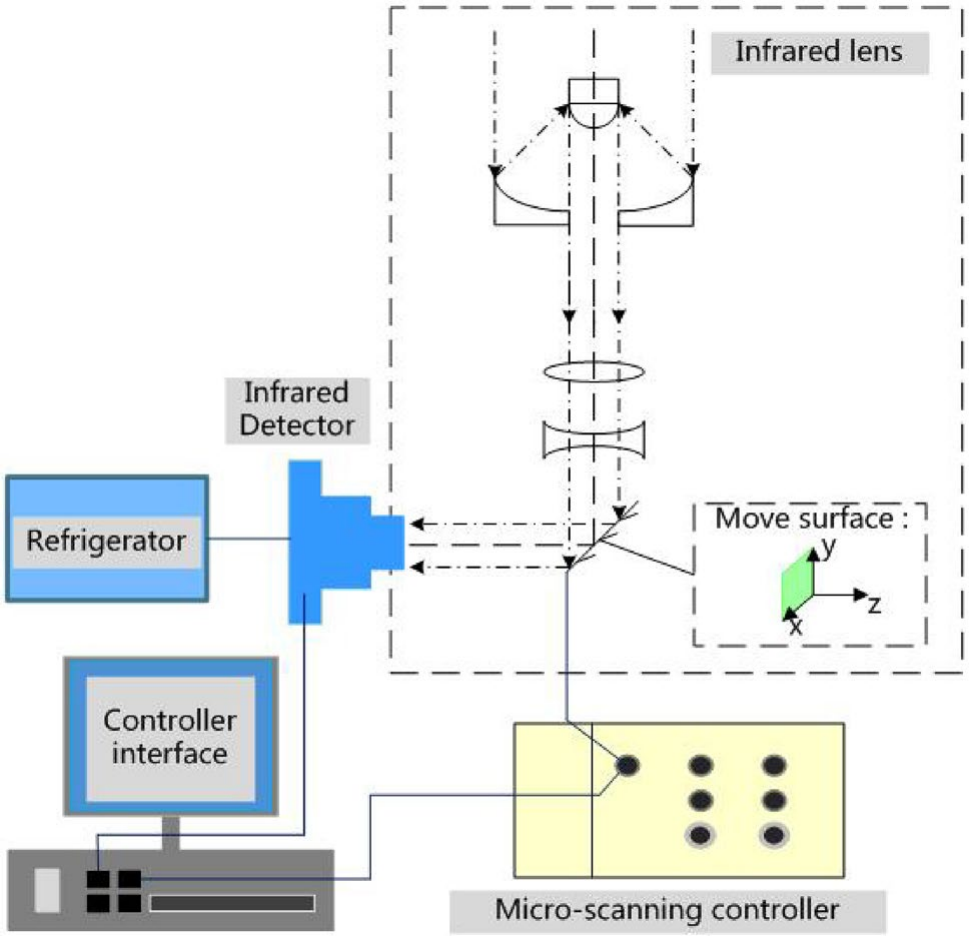
Diagrammatic sketch of reflective infrared micro-scanning optical model.

The diagrammatic sketch of reflective infrared micro-scanning optical model is shown in Fig. 2. The model mainly consists of four parts: infrared lens, micro-scanning controller, refrigeration infrared detector and computer. The high speed galvanometer is connected with micro-scanning controller, and the sub-pixel micro-scanning imaging on the XY plane can be realized by operating micro-scanning control software on the computer.

Aiming at solving the problem of error in the input source of traditional super resolution restoration algorithm, a reflective infrared micro-scanning optical model is proposed. The multi frame low resolution image sequences are generated by using this model. There are no time lap and spatial displacement of the multi frame low resolution image sequences. So the error in the input source of super resolution restoration is eliminated. Furthermore, the combination of infrared micro-scanning optical model and super resolution restoration breaks through the theoretical limit comparing with single imaging algorithm and the performance of super resolution restoration is greatly improved.

## A POCS super resolution restoration algorithm based on region selection (RPOCS)

Considering real-time processing in photoelectric tracking system, this paper introduces threshold segmentation method to distinguish target region and background region. The selection of threshold directly affects integrity of target extraction and restoration effect. If the threshold value is higher, it is easy to classify target to background, so it is unable to extract target region. If the threshold value is lower, it is easy to classify background to target, so false alarm occurs. Traditional Otsu method determines single threshold value according to the maximum^4–7^. The single threshold value is not accurate and the target region cannot be completely separated when the infrared background radiation is strong. Traditional Otsu method is not suitable for infrared dim-small target image. In this paper, an adaptive multi-threshold segmentation method is introduced.

Supposing the infrared image is $f(x,y)$, and its gray level is $\{ L_{0} ,L_{1} ,...,L_{M} \}$. The gray histogram of infrared image is shown as Eq. (1).

1 $$P_{{L_{i} }} = \frac{{n_{{L_{i} }} }}{N}$$

In Eq. (1) $n_{{L_{i} }}$ is pixel number of gray value $L_{i}$ in the whole image, and $N$ is total pixel number in the whole image.

Supposing there are $m$ thresholds $\delta = \{ \delta_{1} ,\delta_{2} ,...,\delta_{m} \}$, such as $L_{0} < \delta_{1} < \delta_{2} < \cdots < \delta_{m} = L_{M}$. These $m$ thresholds divide image region into $m$ categories. For any $\delta_{i} \in \delta$, a segmentation section $[\delta_{i - 1} + 1,\delta_{i - 1} + 2,...,\delta_{i} ]$ can be determined. The probability that pixels of image are in this section is shown as Eq. (2).

2 $$P(\delta_{i} ) = \sum\limits_{{L_{i} = \delta_{i - 1} + 1}}^{{\delta_{i} }} {P_{{L_{i} }} }$$

For the convenience of derivation, we take $m = 3$. For the three sections, the probabilities are shown as Eqs. (3), (4) and (5).

3 $$P(\delta_{1} ) = \sum\limits_{{L_{i} = L_{0} }}^{{\delta_{1} }} {P_{{L_{i} }} }$$

4 $$P(\delta_{2} ) = \sum\limits_{{L_{i} = \delta_{1} + 1}}^{{\delta_{2} }} {P_{{L_{i} }} }$$

5 $$P(\delta_{3} ) = \sum\limits_{{L_{i} = \delta_{2} + 1}}^{{\delta_{3} }} {P_{{L_{i} }} }$$

In Eq. (5), $$\delta_{3} = L_{M}$$.

According to the above probability distribution, the gray mean value $\mu$ of the whole image can be described as Eq. (6).

6 $$\mu = \eta (\delta_{1} ) + \eta (\delta_{2} ) + \eta (\delta_{3} )$$

In Eq. (6), $\eta (\delta_{1} )$, $\eta (\delta_{2} )$ and $\eta (\delta_{3} )$ are shown as Eqs. (7), (8) and (9).

7 $$\eta (\delta_{1} ) = \sum\limits_{{L_{i} = L_{0} }}^{{\delta_{1} }} {L_{i} P_{{L_{i} }} } = P(\delta_{1} )\mu (\delta_{1} )$$

8 $$\eta (\delta_{2} ) = \sum\limits_{{L_{i} = \delta_{1} + 1}}^{{\delta_{2} }} {L_{i} P_{{L_{i} }} } = P(\delta_{2} )\mu (\delta_{2} )$$

9 $$\eta (\delta_{3} ) = \sum\limits_{{L_{i} = \delta_{2} + 1}}^{{\delta_{3} }} {L_{i} P_{{L_{i} }} } = P(\delta_{3} )\mu (\delta_{3} )$$

In Eqs. (7), (8) and (9), $\mu (\delta_{1} )$, $\mu (\delta_{2} )$ and $\mu (\delta_{3} )$ are inner mean value of three sections.

All pixel gray levels are distributed in these three sections, shown as Eq. (10).

10 $$P(\delta_{1} ) + P(\delta_{2} ) + P(\delta_{3} ) = 1$$

In conclusion, it can be solved shown as Eq. (11).

11 $$\mu (\delta_{3} ) = \frac{{\mu - [\eta (\delta_{1} ) + \eta (\delta_{2} )]}}{{1 - [P(\delta_{1} ) + P(\delta_{2} )]}}$$

The energy function $\omega$ is defined as weighted Euclidean distance from three inner mean value $\mu (\delta_{1} )$, $\mu (\delta_{2} )$ and $\mu (\delta_{3} )$ to global mean value of the whole image, shown as Eq. (12).

12 $$\omega = P(\delta_{1} )[\mu (\delta_{1} ) - \mu ]^{2} + P(\delta_{2} )[\mu (\delta_{2} ) - \mu ]^{2} + P(\delta_{3} )[\mu (\delta_{3} ) - \mu ]^{2}$$

The threshold values $\delta_{1}$ and $\delta_{2}$ should be divided as far as possible, that is, maximize the energy function shown as Eq. (13).

13 $$(\delta_{1} ,\delta_{2} ) = \mathop {\arg }\limits_{{L_{i} \in \{ L_{0} ,L_{1} ,...,L_{M} \} }} \max \{ \omega \}$$

Double threshold segmentation is divided into two processes. Firstly threshold value $\delta_{1}$ is used to roughly segment the image. Some target regions will be submerged by background if background radiation is strong. Secondly threshold value $\delta_{2}$ is used to further segment the image. Some target regions will be separated from background region.

For images containing infrared dim-small targets, there is no obvious peak phenomenon in histogram distribution when the contrast between background region and target region is not large. Double threshold segmentation can achieve better segmentation results.

In this paper the process of super resolution restoration for the low resolution image sequences $\{ Lp_{1} ,Lp_{2} ,...,Lp_{n} \}$, firstly the threshold values $\delta_{1}$ and $\delta_{2}$ are determined, secondly the threshold value $\delta_{1}$ is used to segment shown as Eq. (14).

14 $$Lp_{i}^{\prime} (x,y) = \left\{ {\begin{array}{*{20}c} {Lp_{i} (x,y)} & {Lp_{i} (x,y) < \delta_{1} } \\ {255} & {Lp_{i} (x,y) \ge \delta_{1} } \\ \end{array} } \right.$$

In Eq. (14), $Lp_{i}{^{\prime}}$ is low resolution image sequence that segmented with threshold value $\delta_{1}$.

Then threshold value $\delta_{2}$ is used to further segment. Some surplus target regions will be separated shown as Eq. (15).

15 $$Lp_{i}^{\prime\prime} (x,y) = \left\{ {\begin{array}{*{20}c} 0 & {Lp_{i}^{\prime} (x,y) < \delta_{2} } \\ {255} & {Lp_{i}^{\prime} (x,y) \ge \delta_{2} } \\ \end{array} } \right.$$

In Eq. (15), $Lp_{i}{^{\prime}}_{{}}{^{\prime}} (x,y)$ is low resolution image sequence that finally segmented with the threshold value $\delta_{2}$.

After threshold segmentation, the remaining white region $S_{{w_{i} }} (i = 1,2,...,n)$ is considered to be target region. Then target region is overlying shown as Eq. (16).

16 $$S_{w} = S_{{w_{1} }} \cup S_{{w_{2} }} \cup ... \cup S_{{w_{n} }}$$

The white region $S_{w}$ calculated by Eq. (16) contains all target regions in the whole image sequence. In optoelectronic tracking system, we only concern the target region. So, only $S_{w}$ region needs super resolution restoration.

Firstly we determine the minimum circumscribed rectangle $\min {\text{Re}} ct_{top,left}^{w,h}$ of the white region $S_{w}$. $w$ and $h$ are width and height of the rectangle. $(left,top)$ is upper left coordinate of the rectangle. For low resolution image sequences $\{ Lp_{1} ,Lp_{2} ,...,Lp_{n} \}$, new small size low resolution sequences $\{ Lp_{1}^{s} ,Lp_{2}^{s} ,...,Lp_{n}^{s} \}$ is generated from the rectangle of target image.

Secondly for low resolution image sequences $\{ Lp_{1}^{s} ,Lp_{2}^{s} ,...,Lp_{n}^{s} \}$, we take one frame $Lp_{r}^{s}$ for bilinear interpolation as reference frame $Hp_{r}^{s}$. The other frames need image registration and iterative projection correction with reference frame.

Finally we gain high resolution image $Hp^{s}$ of target region.

$Hp_{r}$ is obtained by bilinear interpolation of $Lp_{r}$. $Hp^{s}$ is overlay to $Hp_{r}$. The high resolution image $Hp$ is gained. The background region of $Hp$ is obtained by bilinear interpolation. The target region of $Hp$ is obtained by POCS^8^.

In conclusion, the steps of RPOCS are described as follows.

Step1: The threshold is determined according to Eq. (13). The threshold segmentation is applied to the whole low resolution image sequence according to Eqs. (14) and(15). The target region of each image $S_{{w_{i} }}$ is gained.

Step2: All $S_{{w_{i} }}$ are combined according to Eq. (16). All target regions $S_{w}$ are gained. The minimum circumscribed rectangle $\min {\text{Re}} ct_{top,left}^{w,h}$ is calculated.

Step3: The sub image sequences of target region are extracted to be new low resolution image sequences $\{ Lp_{1}^{s} ,Lp_{2}^{s} ,...,Lp_{n}^{s} \}$.

Step4: A frame of high resolution image is constructed by bilinear interpolation.

Step5: The motion parameters between low resolution image sequences are calculated.

Step6: The low resolution image is mapped onto high resolution grid according to projection operator. Then gray is corrected.

Step7: The exit condition of iteration is judged. Yes, execute Step 8, otherwise, return Step 6.

Step8: $Hp^{s}$ is high resolution region of target region. $Hp_{r}$ is large scale high resolution image constructed by bilinear interpolation. $Hp$ is final high resolution image.

**Figure 3:**
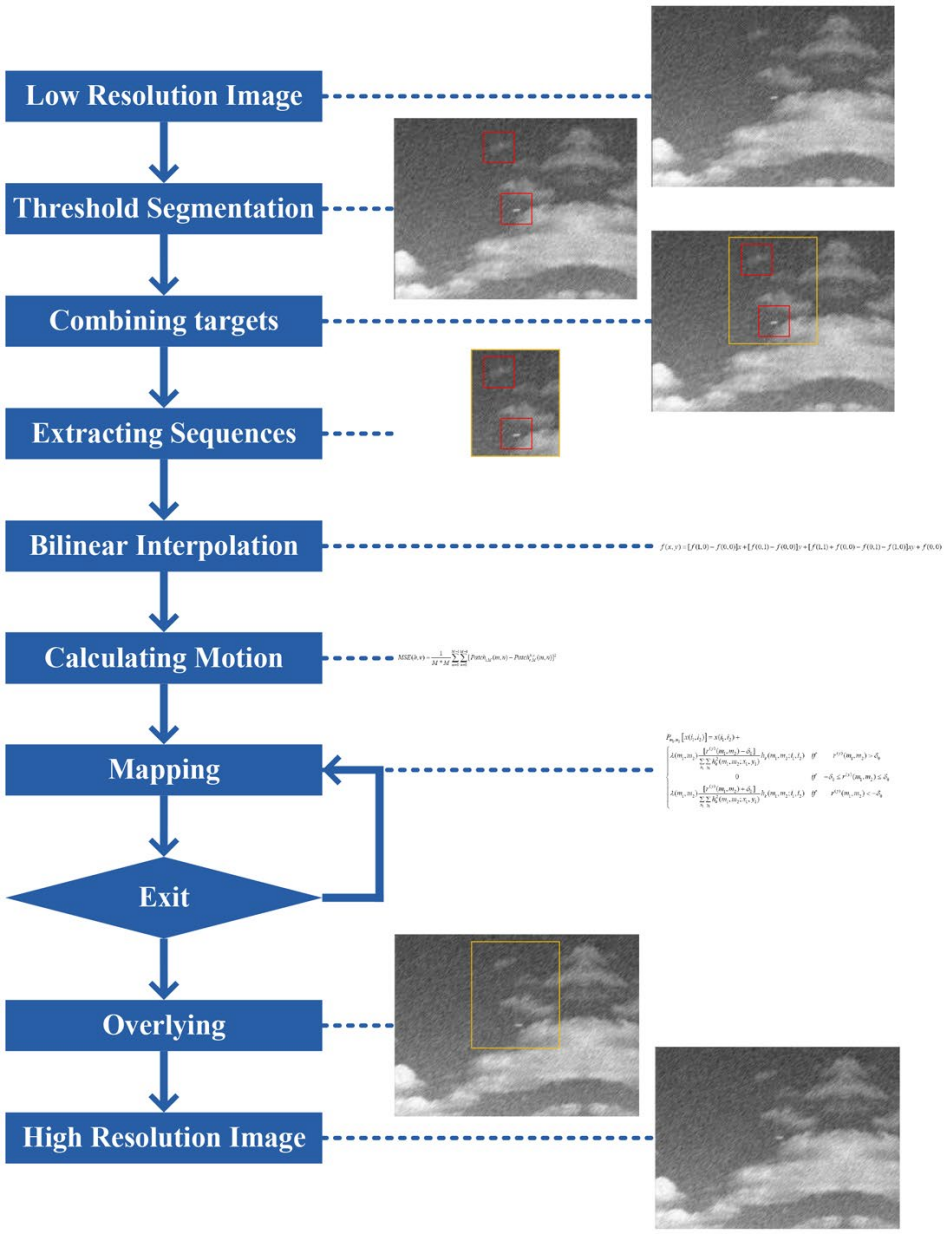

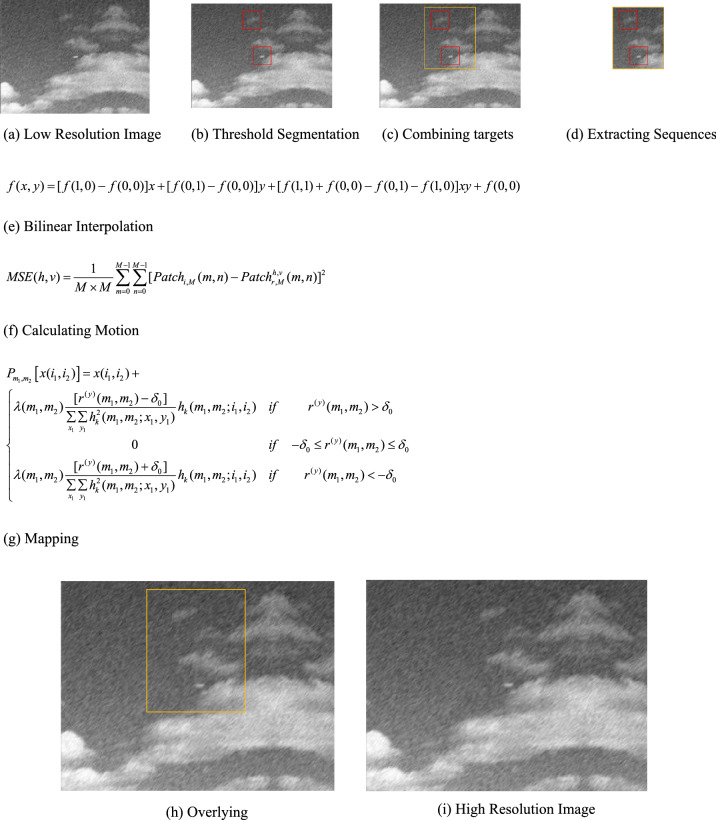
Flow chart of RPOCS (the detail magnification of this figure is show below from **(a)–(i))**

The flow chart of RPOCS is shown in Fig. 3.

## Experimental evaluations of RPOCS based on reflective infrared micro-scanning optical model

**Figure 4:**
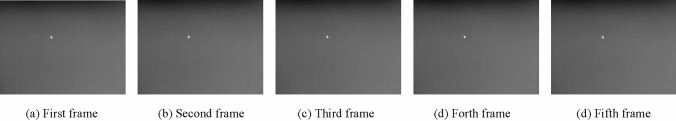
Low resolution image sequences of plane image in sky background.

According to infrared micro-scanning optical model built in Fig. 2, the simulated infrared camera uses a resolution of 320 × 240 for experimental verification. Background is sky. The resolution of plane image in sky background is 320 × 240. The plane images in sky background are shown in Fig. 4.

**Figure 5:**
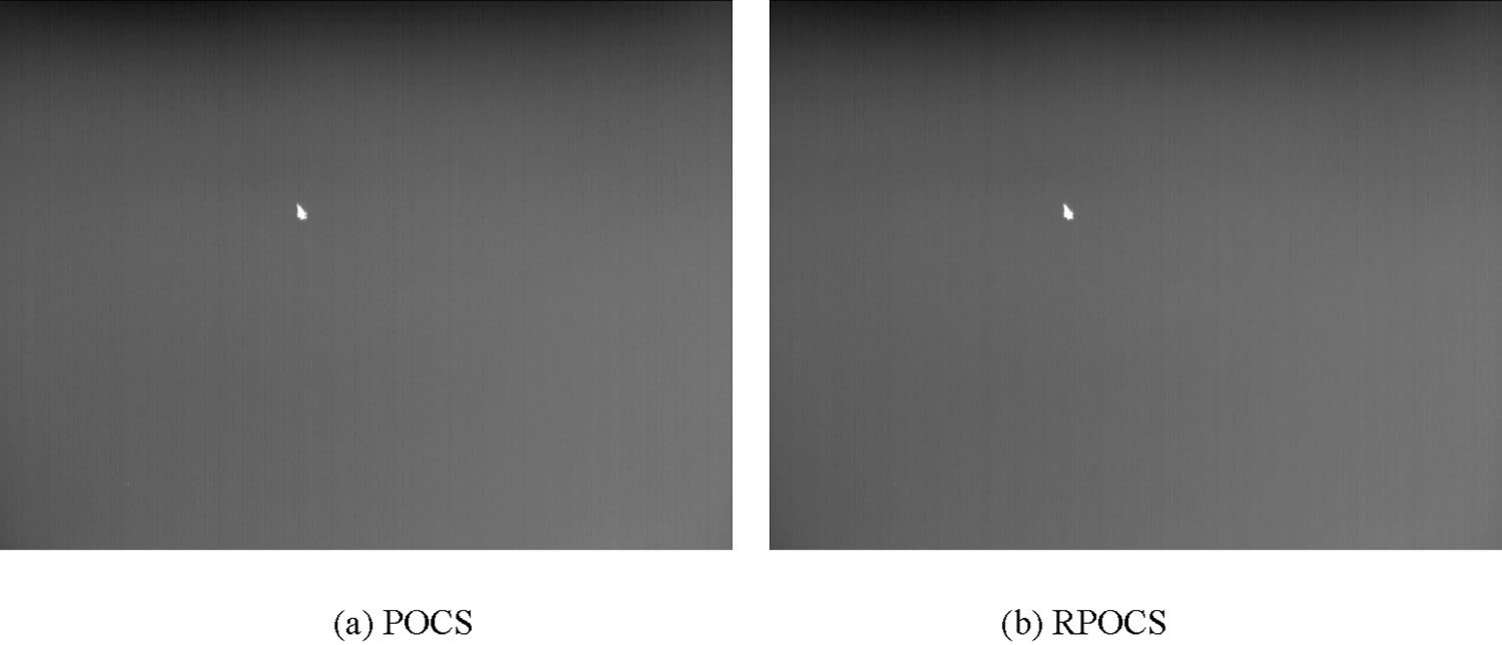
Effect of two restoration algorithms for plane image in sky background.

The restoration resolution of plane image in sky background is 640 × 480. The effect of two restoration algorithms for plane image in sky background is shown in Fig. 5.

From Fig. 5a, it can be seen that traditional POCS restoration algorithm has strong noise in both background and target region. It shows that traditional POCS algorithm has poor anti noise ability, and eve noise amplification occurs locally. At the same time, the outline of target is also fuzzy. The restoration effect of POCS is not ideal.

From Fig. 5b, it can be seen that RPOCS restoration algorithm also has large noise in background. This is because RPOCS only restores target region and bilinear interpolation is used to background region.

POCS and RPOCS are compared by SSIM_NCCDFT^9^ (Structural SIMilarity Normalized Cross Correlation of DFT) and processing time. The SSIM_NCCDFT and processing time of plane image in sky background are shown in Table 1.

**Table 1:** SSIM_NCCDFT evaluation contrast and processing time of the two restoration algorithms.

Method Contrast	POCS	RPOCS
SSIM_NCCDFT	0.9641	0.9644
Processing time(s)	3.597	0.045

From Table 1, it can be seen RPOCS has better performance than traditional POCS by using SSIM_NCCDFT for evaluation. From the overall effect, RPOCS has better performance than traditional POCS. From the processing time, it can be seen RPOCS is further faster than traditional POCS. This is because RPOCS only restores target region and traditional POCS restores whole image. The processing time of RPOCS is 1% lower than traditional POCS. The traditional POCS is poor on noise suppression and edge detail retention. The processing time is longer. Therefore it is inapplicable for photoelectric tracking system. RPOCS uses bilinear interpolation in background and only restores target region. So the processing time is faster. So it is suitable for photoelectric tracking system.

## Conclusions

Aiming at solving the problem of error in the input source of traditional super resolution restoration algorithm, a reflective infrared micro-scanning optical model is proposed. The multi frame low resolution image sequences are generated by using this model. There are no time lap and spatial displacement of the multi frame low resolution image sequences. So the error in the input source of super resolution restoration is eliminated. Furthermore, the combination of infrared micro-scanning optical model and super resolution restoration breaks through the theoretical limit comparing with single imaging algorithm and the performance of super resolution restoration is greatly improved.

For the long iteration of POCS and the shortcomings of incapability to meet real-time detecting of optical detection system, a POCS super resolution restoration algorithm based on region selection is proposed. The target region is the key point that we focus on in the optical detection system, while this region contains only very small number of pixels. Therefore, we use threshold segmentation and combination to acquire the union of all targets regions. Then we execute super resolution restoration only in the union of all targets regions. In this way we decrease huge computation of background restoration and greatly reduce the operation time to achieve real-time or near real-time. So this super resolution restoration algorithm can be applied in the practical infrared image processing system.
